# Action Planning and the Timescale of Evidence Accumulation

**DOI:** 10.1371/journal.pone.0129473

**Published:** 2015-06-12

**Authors:** Konstantinos Tsetsos, Thomas Pfeffer, Pia Jentgens, Tobias H. Donner

**Affiliations:** 1 Department of Experimental Psychology, Oxford University, 9 South Parks Road, Oxford, OX1 3UD, United Kingdom; 2 Department of Psychology, University of Amsterdam, Weesperplein 4, 1018 XA, Amsterdam, The Netherlands; 3 Amsterdam Brain and Cognition, University of Amsterdam, Nieuwe Achtergracht 129, 1018 WS, Amsterdam, The Netherlands; 4 Department of Neurophysiology and Pathophysiology, University Medical Center Hamburg- Eppendorf, 20246, Hamburg, Germany; 5 Netherlands Institute for Neuroscience, Meibergdreef 47, 1105 BA, Amsterdam Zuidoost, The Netherlands; 6 Bernstein Center for Computational Neuroscience, Charitein Center for Comput, Haus 6, Philippstrast 13, 10115, Berlin, Germany; University of Sheffield, UNITED KINGDOM

## Abstract

Perceptual decisions are based on the temporal integration of sensory evidence for different states of the outside world. The timescale of this integration process varies widely across behavioral contexts and individuals, and it is diagnostic for the underlying neural mechanisms. In many situations, the decision-maker knows the required mapping between perceptual evidence and motor response (henceforth termed “sensory-motor contingency”) before decision formation. Here, the integrated evidence can be directly translated into a motor plan and, indeed, neural signatures of the integration process are evident as build-up activity in premotor brain regions. In other situations, however, the sensory-motor contingencies are unknown at the time of decision formation. We used behavioral psychophysics and computational modeling to test if knowledge about sensory-motor contingencies affects the timescale of perceptual evidence integration. We asked human observers to perform the same motion discrimination task, with or without trial-to-trial variations of the mapping between perceptual choice and motor response. When the mapping varied, it was either instructed before or after the stimulus presentation. We quantified the timescale of evidence integration under these different sensory-motor mapping conditions by means of two approaches. First, we analyzed subjects’ discrimination threshold as a function of stimulus duration. Second, we fitted a dynamical decision-making model to subjects’ choice behavior. The results from both approaches indicated that observers (i) integrated motion information for several hundred ms, (ii) used a shorter than optimal integration timescale, and (iii) used the same integration timescale under all sensory-motor mappings. We conclude that the mechanisms limiting the timescale of perceptual decisions are largely independent from long-term learning (under fixed mapping) or rapid acquisition (under variable mapping) of sensory-motor contingencies. This conclusion has implications for neurophysiological and neuroimaging studies of perceptual decision-making.

## Introduction

A hallmark of perceptual decision-making is the integration of evidence for different states of the world [1]. Imagine driving your car on a rainy day and reading a street sign to decide whether to turn left or right. Since the “sensory evidence” you are trying to interpret is noisy (i.e., it fluctuates randomly), you can improve your judgment by integrating evidence over time [1,2,3].

The timescale of this integration process is a key psychophysical parameter quantifying perceptual decision-making as it reflects the network mechanisms underlying the accumulation of sensory information in the brain [4–7]. While many studies of non-sensory (top-down) effects in perceptual decision-making have focused on strategic adjustments of the decision threshold that terminates the decision process [3,6,8,9,10], only few previous studies have investigated direct top-down effects on the evidence integration process *per se*, as indicated by the integration timescale [11–14]. Only two of these studies were conducted in human observers [11,13] whose integration mechanisms may differ from those of other species [15]. Here, we examined the effect of one important top-down factor that has not been previously examined: knowledge of sensory-motor contingencies.

In the above example, the integrated evidence is continuously mapped onto a plan to select and execute a motor movement. The same holds for most previous neurophysiological laboratory studies of perceptual decision-making [1,16,17,18]. Under such conditions, build-up signatures of evidence integration are found in brain regions involved in action planning. In particular, when perceptual choices are reported as saccades [12,19,20] or hand movements [21,22] choice-specific activity ramps up in the corresponding (pre-)motor brain regions. These premotor build-up signatures are not evident if the sensory-motor contingencies are broken up by instructing the mapping between perceptual choice and motor response only after stimulus presentation [12], or by eliminating the motor response altogether (in a covert counting task) [23]. This raises the question whether knowledge about sensory-motor contingencies might also improve (i.e., prolong) integration timescales observed behaviorally. Further, it has been shown that learning of fixed sensory-motor contingencies (over hundreds of trials or more) improves the selectivity of the read-out of sensory information by the association cortex [24,25]. But it remains unknown whether such learning also improves the integration timescale.

We addressed these questions in six human observers performing the same motion discrimination task under three different sensory-motor mapping conditions. In one experiment, the mapping between decision outcome and motor response varied on a trial-by-trial basis. In different conditions, this mapping was instructed before or after stimulus presentation. While the first condition allowed the integration of evidence directly towards action plans, the second did not. We found that integration timescales were generally shorter than the maximum stimulus duration, and thus shorter than the timescale required to maximize the fraction of correct choices in the task. But integration timescales were indistinguishable between conditions. We then reasoned that sensory-motor mapping might only improve integration timescales if fixed over many trials, due to a slow learning process. Thus, we asked the same observers to perform the task under fixed mapping in another experiment. Again, despite extensive practice, integration timescales were indistinguishable from the other two conditions. We conclude that the integration of perceptual evidence does not depend on sensory-motor contingencies.

## Materials and Methods

### Ethics Statement

The ethical committee of the University of Amsterdam approved the study (reference number 2011-OP-1588). Written informed consent was obtained from all participants.

### Observers

Six healthy human observers were recruited for this study (2 males, mean age: 25; range: 22–29 years). All observers had normal or corrected-to-normal vision. The pool included four observers who were naïve with respect to the purpose of the experiment, and two authors (P.J. and T.P). Observers received either course credits or were paid a small amount of money (€10/hour) for their participation.

### Stimuli

We used an established psychophysical approach for quantifying the perceptual evidence integration timescale [12,13,26–29], which entailed the following two aspects of the sensory input: First, we systematically manipulated the duration of the stimulus (i.e., the maximal evidence integration time available to the observer) and prompted the response after that time (“interrogation protocol”). Second, we systematically manipulated the strength of the perceptual evidence to estimate the observer’s perceptual discrimination threshold for each stimulus duration. This enabled us to quantify discrimination thresholds as function of stimulus duration (see below).

Using this general approach, we performed two experiments. Below, we first describe all general aspects, followed by the specifics of each experiment. Motion stimuli consisted of “random dot kinematograms” (RDK), consisting of 785 white dots (on average) within a circular aperture 9.1° in diameter (dot density: 12.07 dots per deg^2^), centered on a red fixation cross (0.4° x 0.4°), and displayed against a black background. Individual dots subtended 0.04° x 0.04°. On each frame, the dots were randomly assigned to either a population of “signal dots” or of “noise dots”. The signal dots were randomly selected on each frame and were displaced from frame to frame with a fixed spatiotemporal offset, creating a coherent motion signal with upward or downward direction (separated by 180°) and a speed of 2.6°/s. We used “random position” noise. That is, the noise dots were re-drawn on a randomly selected position, creating spatiotemporal white noise, which comprised a mixture of directions and speeds [30]. On each trial, three different “sets” of RDKs of the selected direction and coherence were plotted in an interleaved fashion, where the dot pattern from each set was shown for one frame and followed by the next pattern from the same set only after three successive video frames, and so forth. This version of the RDK stimulus corresponds to the one used in many of the seminal monkey physiology studies on temporal integration of visual motion information (e.g. [31]). This was to encourage integration of motion information across space and time. The percentage of coherently moving dots (“motion coherence”; 0.05, 1.26, 3.15, 7.92, 19.91, and 50%), viewing duration (150, 300, 600, 1200, 2400, and 4800 ms), direction (“up/ down”), and “decision-to-response-mapping” (“DR-mapping”; e.g. upward motion left button and downward motion right button) were randomly chosen on each trial, under the constraint that each combination of these parameters occurred equally often within a block of 144 trials. The six coherence levels listed above were determined in extensive pilot sessions, tailored to sample the full psychometric function for all stimulus durations. New stimuli were generated for each experimental block, including six different variations of the interleaved RDK sequences.

Stimuli were displayed on a 22-inch CRT monitor (resolution: 800 x 600 pixels) at a rate of 100 Hz. The viewing distance was 68 cm. Experiments were conducted in a dimly illuminated room. Subjects were seated in an adjustable chair with their chin resting comfortably in a chin cup and additional support was provided by a head restraint mounted on the table. The height of the monitor placed the center of the display at approximately eye level.

### Task and procedure

Throughout all experimental conditions, subjects were required to fixate the red cross in the center of the screen and classify the net motion in a stimulus as upward (50% of trials) or downward motion by pressing one of two buttons (left or the right index finger) when a response prompt was provided. The RDK presentation was followed by a variable delay interval, after which the response was prompted. Observers were under no time pressure to respond. Auditory feedback of 100 ms duration (a 1000 Hz tone) was provided for incorrect responses.

#### Variable DR-mapping experiment

**Fig 1:**
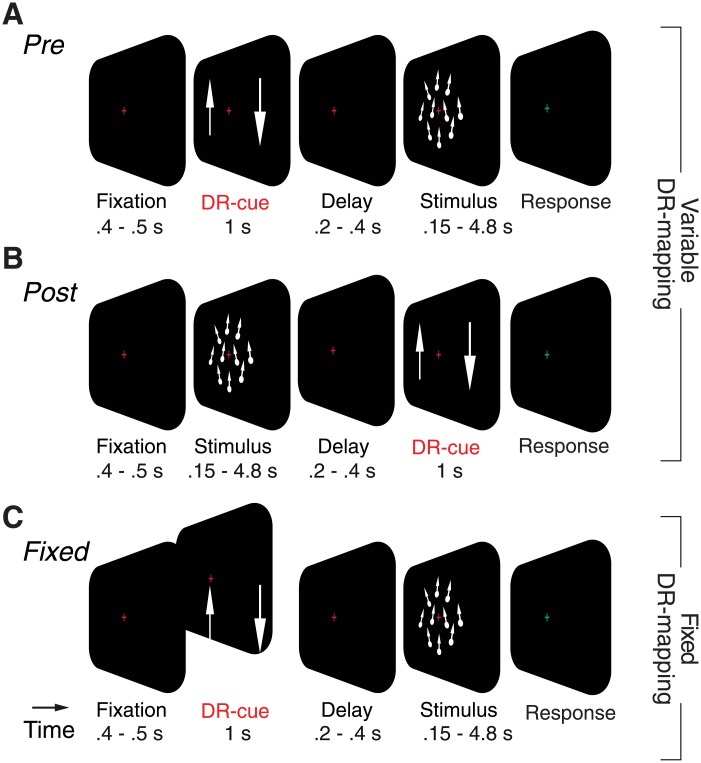
Experimental design. On each trial, the observer was required to discriminate the direction (upward or downward) of the random dot kinematogram (RDK), while fixating the central crosshair. The RDK was presented for one of a number of different durations and levels of motion strength **(A)** Variable “Pre” DR-mapping condition. The DR-mapping cue (two arrows mapping up/down motion directions onto left/right hand button presses) was presented before the RDK (separated by a variable delay), and it varied randomly from trial to trial. After another variable delay, a color switch of the fixation cross prompted the observer to indicate the choice with a button press. **(B)** Variable”Post” DR-mapping condition. Identical to “Pre”, except that the DR-mapping cue was presented after the RDK (“Post”). **(C)** “Fixed” DR-mapping condition. Identical to “Pre”, except that the DR-mapping cue was kept constant throughout the experiment, enabling long-term learning of sensory-motor associations.

The decision-response mapping (e.g., left-hand button press for indicating “upward” choice) varied randomly from trial to trial. We will henceforth abbreviate this as “DR-mapping”. The experiment consisted of two conditions, which differed only in the timing of the cue instructing observers about the DR-mapping, relative to presentation of the RDK stimuli (Fig 1A and 1B). The cue indicated the motion direction corresponding to each response button in terms of two white arrows (one upward, one downward pointing) presented on the left and right half of the screen (11° from fixation).

In the pre-cueing (“Pre”) condition (Fig 1A), the arrows were presented after a variable period of fixation for 1000 ms, and were followed by a second random delay (200–400 ms) and the onset of the motion stimulus (150–4800 ms). After stimulus offset and another random delay (200–400 ms) the fixation cross turned green, which prompted the subject to report their choice.

The post-cueing (“Post”) condition was identical, except that DR-mapping cue was presented after the motion stimulus and second delay (Fig 1B). In both conditions, the inter-trial intervals were 900 ms. “Pre”- and “Post”-conditions were conducted in alternating blocks of 144 trials (see *General design* below).

#### Fixed DR-mapping experiment

This experiment consisted of a single condition, which was identical to the “Pre”-condition, with the exception that the DR-mapping was kept constant across all trials (Fig 1C). Although the DR-mapping was instructed at the start of the experiment and remained constant thereafter, the DR-cue was shown at the beginning of each trial to keep the visual input and trial duration identical to the “Pre”- from the variable DR-mapping experiment. Each subject was first trained on the task for a minimum of 432 trials and then completed between 2448 and 7632 trials (distributed over 3–9 experimental sessions), which were used for the analyses reported in this paper.

#### General design

All statistical analyses reported in this paper were performed within individual observers. Given the large number of trials required from each observer per condition (minimum: 2016) and the clear effect evident in Subjects 1–3 who participated in all conditions (see Results), the remaining three observers were asked to only participate in a subset of conditions. Subjects 4 and 5 were used to replicate the comparison between “Pre” and “Post”. Subject 6 was used to replicate the comparison between the variable and “Fixed” mappings.

The experimental conditions were arranged as follows. Subjects 1–5 first performed the Variable DR-mapping experiment, in which they alternated between “Pre” and “Post” conditions in a pseudorandom order. Each experimental session consisted of 6 blocks: 3 blocks of each condition (“Pre” and “Post”). Subjects 1–3 then performed the Fixed DR-mapping experiment. General order effects would have (if anything) predicted an improvement (prolongation) of integration timescales in the “Fixed” condition, due to the extensive practice of the task (under variable and fixed mapping). By contrast, we found no change of integration timescale in these subjects. Nonetheless, to rule out any order effects, we flipped the order in one additional observer (subject 6), who started with “Fixed”, followed by “Post”. Each subject was first trained on the task for a minimum of 432 trials per condition. Subjects then completed between 2110 and 5760 trials of each condition (distributed over 3–7 experimental sessions in total).

### Model-independent analysis of integration timescale

#### Quantifying psychophysical thresholds

**Fig 2:**
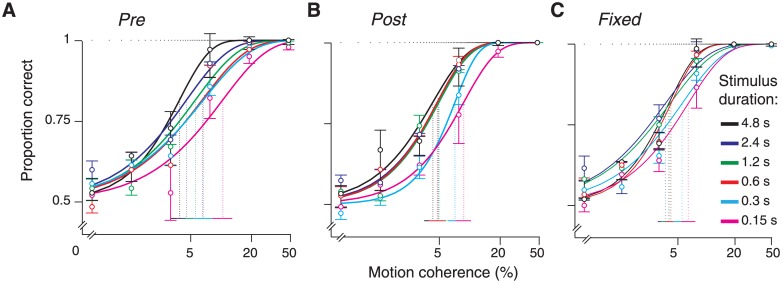
Example psychometric functions of one observer in all conditions. Solid curves are maximum likelihood fits of cumulative Weibull functions to the proportion correct data. Vertical dashed lines represent estimated threshold parameters (solid horizontal lines at bottom, 95% confidence intervals). The horizontal dashed line represents the lapse rate. **(A)** “Pre”- condition. The performance data and psychometric functions are shown separately for all stimulus durations. **(B)** As in A, but for “Post”. **(C)** As in A, but for “Fixed”.

Given the task design (interrogation protocol with different levels of stimulus strength), our analyses focused on proportion correct data, rather than response times [3]. We fitted the observers’ proportion of correct choices as a function of motion coherence, denoted P(C) below, by means of a cumulative Weibull function [32], separately for each experimental condition and stimulus duration. See Fig 2 for example fits from one subject in both conditions of Experiment 2. The cumulative Weibull distribution function was defined as

$$P_{t_{1}\leq t\leq t_{2}}\left(C\right)\;=\;0.5+\left(0.5-\lambda \right)\left(1-exp\left[-({\frac{C}{a})}^{\beta }\right]\right)$$ (1)

where *λ*, *α* and *β* are free parameters and the value 0.5 represents chance performance. The lapse rate (λ) represents stimulus-independent errors, corresponding to the fraction of incorrect choices at the highest motion strength and longest viewing duration. *λ* was determined by fitting Eq (1) to data from the longest viewing duration in each experiment, and then inserted in Eq 2 to find best fits of *α*, and *β* to the data from all conditions. The value of *α* is the psychophysical threshold corresponding to the coherence level that elicits 82% correct responses when asymptotic performance is perfect (i.e., *λ = 0*). Parameter *β* determines the steepness of the psychometric function for a particular threshold. Best-fitting values for the free parameters were obtained by means of a maximum likelihood procedure [33]. To obtain the best-fitting values for the free parameters, we minimized the negative log-likelihood, which yields the exact same parameters as maximizing the likelihood.

Confidence intervals for the proportion of correct choices were obtained by employing a non-parametric bootstrap [34], in which the original set of trials was resampled with replacement a large number of times (N = 10,000) and the proportion of correct responses was computed for each iteration. The confidence intervals of the parameters of the cumulative Weibull functions and of the regression-based threshold vs. duration functions (see below, eqs 2–5) were obtained by means of a parametric bootstrap procedure [35]. We used a binary process to generate a new set of data based on the binomially distributed noise and estimated Weibull parameters from the observed data set. We repeated the maximum likelihood procedure for each bootstrap iteration to find the best parameter fits for the “mock” data set and calculated the corresponding parameters anew. The resulting distributions indicated the likely spread of all parameters for the original data set.

#### Fitting threshold versus duration functions

Perfect integration predicts a linear decrease in threshold with duration with a slope of -0.5 in log-log coordinates. A lack of integration predicts a flat line (slope of 0). Hence, to analyze the dependence of thresholds on duration, we fitted a bilinear function to the log of the best fits of *α* and viewing duration [26–28]. The slope of the first line was constrained to -0.5 and the slope of the second line was constrained to 0. We determined the best fitting value for the intercept *β*_0_ of the linear function. The general fit was evaluated by calculating the sum of squared errors (SSE) and the best fit of the bilinear function was determined by means of an iterative least squares method [36].

With a number of durations *n*, the relationship between thresholds and viewing duration for each *i* = 1,…*n* was expressed as

$$y_{i}\;=\;\beta_{0}-0.5\text{log}\left(t_{i}\right)+e_{i},\;if\text{log}\left(t_{i}\right)\leq A_{i}$$

and

$$y_{i}\;=\;{\beta '}_{0}+e_{i},\;if\text{log}\left(t_{i}\right)>A_{i}$$ (2)

where *β*_0_ correspond to the *y*-intercept of the first line, ${\beta '}_{0}$ corresponds to the *y*-intercept of the second line, *A* represents the abscissa of the joint point. In order to ensure that the two lines join at the value *x* = *A*, we applied the following restriction:

$$\beta_{0}-0.5A\;=\;{\beta_{0}+e}_{i}$$ (3)

The error terms (i.e. *e*_*i*_’s for *i* = 1,…*n*) were independent and identically distributed normal random variables with mean equal to zero and constant variance. Due to the restriction from Eq (3), the abscissa of the joint point could be estimated as

$$A\;=\;\frac{\left(\beta_{0}-{\beta '}_{0}\right)}{0.5}$$ (4)

Eq (4) held only for cases in which *A* lied between two consecutive values of log(*t*), log(*t*_*j*_) and log(*t*_j+1_). In this case, the first line included *n*_*j*_ number of log(*t*_*i*_) and log(*a*_*i*_) pairs up to and including log(*t*_*j*_) and log(*a*_*j*_), whereas the second line included all values from log(*t*_j+1_) and log(*a*_j+1_) on to $\;{n'}_{j}$. If the estimated joint point was indeed log(*t*_*j*_), and log(*t*_j+1_), we computed the ordinary least squares solutions and determined the joint point by substituting the observed *β*’s with the estimated *β*’s. The SSE was computed as the sum of the individual SSEs from the two lines

$$SSE_{j}\;=\;\sum_{i\;=\;1}^{j}{\left[\text{log}\left(a_{i}\right)-(\beta_{0}-0.5log(t_{i}))\right]}^{2}+\sum_{i\;=\;j+1}^{n}{\left[\text{log}\left(a_{i}\right)-{\beta '}_{0}\right]}^{2}$$ (5)

If the estimate of the joint point was not between log(*t*_*j*_), and log(*t*_j+1_), we modified the computation such that *A* occurred exactly at log(*t*_*j*_). Since the minimization of SSE in this case is constrained to only one possible joint point log(*t*_*j*_), this constrained least squares solution was computed as a modification of the two separate ordinary least squares solutions [36].

In a control analysis, we relaxed the constraints on the slopes of the bi-linear fits, by allowing the first slope to be in the range from -0.5 to 0, and the second slope was constrained to any value larger than the first slope. These fits required two additional constraints. 1. If the estimated joint point exceeded the longest signal duration in the experiment, it was set to the longest signal duration (4.8 s). 2. If the first slope was smaller than 0.001, we assumed that participants did not integrate and set the joint point to the shortest signal duration of 0.15 s.

**Fig 3:**
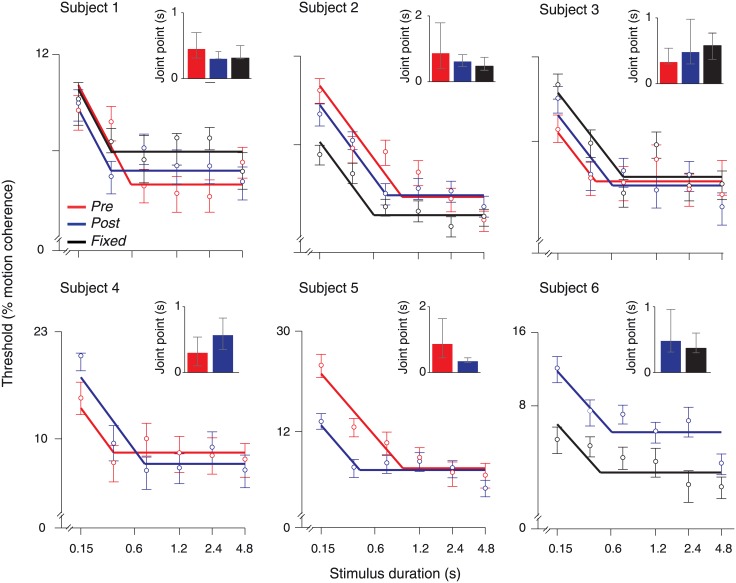
Model-independent characterization of integration timescales. Threshold vs. duration functions from all conditions. Circles represent psychophysical thresholds for each stimulus duration. Solid lines: best fitting bilinear function, with the slopes constrained to -0.5 (first branch) and 0 (second branch; see text for details). Error bars, 60% confidence intervals (bootstrap). Inset: bar graphs of the joint point estimates of the best fitting bilinear function. Error bars, 95% confidence intervals (bootstrap).

Given the six stimulus durations, the joint point could only be estimated for the four intermediate durations, which corresponded to the limited interval 0.3–2.4 s. We verified that in all cases, the bi-linear fits provided a significantly better match to the data than a single linear fit, with or without slopes constrained to -0.5 (data not shown). Further, in all cases were the joint point estimates significantly shorter than the longest possible timescale estimates of 2.4 s (see Results, Fig 3). Thus, it is unlikely that the short timescale estimates obtained are due to the limitations of the procedure.

#### Statistical comparisons of joint points

To compare integration time scales and lapse rates between conditions within individual observers (i.e., “Pre”- versus “Post”-conditions; “Post” vs. “Fixed”; “Post” vs. “Fixed”), we compared the joint point estimates *A* by means of non-parametric permutation tests [34]. For each permutation, all trials were combined into one set of N_pre_/N_fixed_ + N_post_ trials and shuffled 10,000 times. Then the shuffled set was split into 2 sets of N_pre_/N_fixed_ and N_post_ trials, the proportion correct was recalculated and the Weibull functions were fitted to determine the new thresholds and lapse rates. Based on these, *A* was iteratively computed to obtain the permutated difference between the two sets. Finally, we compared the observed difference lapse rates and in *A* with the permutated differences. P-values were obtained by calculating the fractions of repetitions (10,000), in which the absolute value of the permutated difference was larger than the absolute value of the measured difference.

### Computational modeling of psychophysical performance

#### General model description

We fitted the leaky-competing accumulator model (LCA) [37,38] to the behavioral data of each individual observer. The LCA is a neurophysiologically inspired sequential sampling model of decision-making and has successfully accounted for behavioral data from a wide variety of perceptual tasks [2]. Similar to other models, LCA assumes that noisy evidence for different hypotheses is accumulated towards a decision criterion. A separate accumulator, corresponding to the pooled neuronal activity of a dedicated population, represents each competing hypothesis. Thus, in choices between two options, two pools of neurons integrate the momentary evidence for each alternative and compete with each other via lateral inhibition, while their activity is subject to slow decay (“leak”). The activation states of the accumulators are described by the following finite difference equations:

$$\begin{array}{c} x_{1}\left(t+1\right)\;=\;max\left(0,x_{1}\left(t\right)+\Delta x_{1}\right);\\ x_{2}\left(t+1\right)\;=\;max\left(0,x_{21}\left(t\right)+\Delta x_{2}\right);\\ \Delta x_{1}\;=\;I_{1}-\kappa x_{1}-\beta x_{2}+I_{0}+N\left(0,\sigma \right);\\ \Delta x_{2}\;=\;I_{2}-\kappa x_{2}-\beta x_{1}+I_{0}+N\left(0,\sigma \right). \end{array}$$ (6)

In the above equation *x*_*i*_ corresponds to the activation states of the accumulator associated to alternative *i*. The *max* function prevents activation states (which correspond to population firing rates) from going below a predefined value, implementing in this case a lower reflecting boundary at 0. Constant input to both accumulators (controlling for the degree of non-linearity in the activation states) is denoted by *I*_0_ while Δ is the momentary change of each accumulator on each time step. This change or increment is driven by three factors: i) the external input in favor of the corresponding accumulator, ii) the activation state of the accumulator on the preceding moment and iii) the activation state of the competing accumulator. The external momentary input, denoted by *I*_*i*_, is subject to Gaussian noise fluctuations with zero mean and standard deviation *σ*. The accumulators compete with each other via lateral inhibition of strength *β*, and their activation is subject to leak of κ. For the interrogation protocol used in this study, the activation states of the two accumulators are read out at the end of the trial (corresponding to the response cue presentation), and the alternative with the highest activation state, up to that point, is chosen.

In the simulations presented here, we fixed the standard deviation of noise to σ = 1. We further assumed that *I*_1_ = *C* × *s*, with *C* corresponding to the motion coherence for the corresponding alternative and *s* being the sensitivity parameter that modulates the signal to noise ratio. We let *I*_0_ be a free parameter, and we assumed that motion coherence is subject to power-law saturation with exponent *m*: *I*_1_ = *C* ^*m*^ × *s*. The experimental time units were converted into simulation time steps with 1 second corresponding to 250 time-steps. The coherence level (*C*) was determined by the experimental condition and ranged from 0 (no coherent motion) to 1 (all dots move coherently towards a given direction). The input to the first unit (*I*_1_) was always proportional to the coherence level *c*, while the input to the second unit (*I*_2_) was always set to 0. In sum, the decision-making model had five free parameters: inhibition (*β*), leak (κ), sensitivity (*s*), constant activity (*I*_0_) and coherence saturation (*m*).

#### Model fits to behavioral data

We fitted the model described above to the data of each individual observer. Specifically, the model was fitted to the individual proportion correct data for *N* coherencies x *M* duration levels (*N* = 5 and *M* = 6). We excluded the lowest coherence level (0.05) because performance indistinguishable from chance for all durations at that coherence and we found that including this coherence yielded worse fits. Assuming that the correct responses follow a binomial distribution, we computed the likelihood for a given parameterization of the model, for the *K* = *N x M* data points as:

L = ∏ikniyipiy(1-pi)ni-yi (7)

where *n*_*i*_ was the number of trials for the *i-th* data point, *y*_*i*_ was the corresponding number of correct responses and *p*_*i*_ the probability of correct response predicted by the model (obtained by running 5000 iterations of the model for the given condition/ parameterization). The cost function was the negative logarithm of L:

$$-LL\;=\;-{log}_{e}(L)$$ (8)

and was minimized using SUBPLEX minimization routine [39]. For each subject and each model we ran the optimization 400 times with starting points randomly sampled from uniform distributions within a parameter-specific range.

In order to assess the goodness of fit for the best parameters of a given model, we calculated the chi-square statistic as follows:

$$\chi^{2}\;=\;\sum_{i\;=\;1}^{K}\frac{{\left(O_{i}-E_{i}\right)}^{2}}{E_{i}}$$ (9)

where *K* was the number of bins corresponding to the experimental conditions, *O*_*i*_ was the observed frequency of correct responses at condition *i*, and *E*_*i*_ the corresponding frequency predicted by the model. Because the number of experimental and simulated trials was different, *E*_*i*_ was calculated by multiplying *p*_*i*_ (the probability of correct response predicted by the model) by the number of experimental trials. The chi-square statistic had *K*-1 degrees of freedom. P-values indicated the probability that the chi-square statistic is at least as extreme as the obtained one, under the null hypothesis that the data and the predictions of the model follow the same distribution. We rejected the null hypothesis at a significance level of *α* = 0.05.

#### Model-based estimation of integration time constants

**Fig 4:**
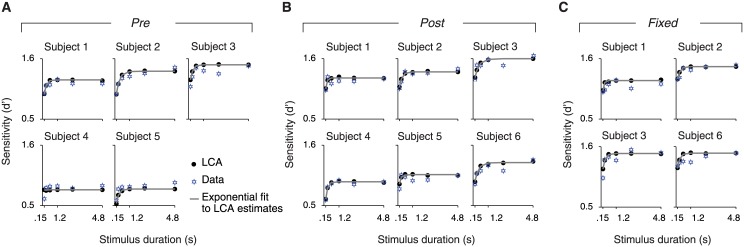
Model-based characterization of integration timescales. Simulated d’ vs. duration functions, and exponential fits. Filled dots are the best-fitting LCA model estimates for each subject; solid curves: shifted exponential functions on the predicted average sensitivity across all coherences; blue stars: measured average sensitivity across all coherences. **(A)** “Pre”. **(B)** “Post”. **(C)** “Fixed”.

**Fig 5:**
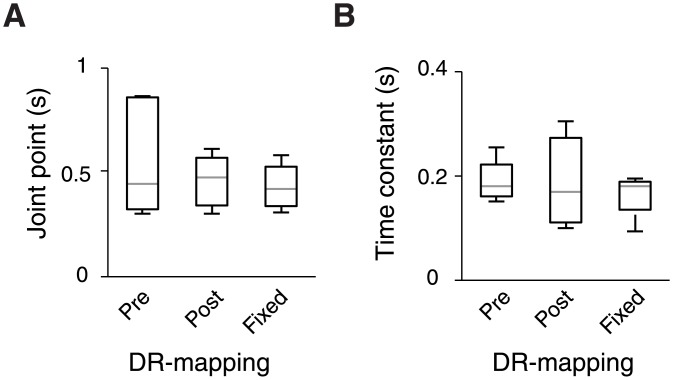
Summary of integration times using model-independent (A) and model-based (B) characterization of time-constants. Summary of integration times under the three DR-mappings tested. The gray horizontal line marks the median, the upper and lower edges of the box mark the 25^th^ and 75^th^ percentiles, and the whiskers extend to the most extreme data points, excluding outliers. **(A)** Joint points of the bilinear fit to threshold vs. duration functions. **(B)** Time constants derived from LCA model fits.

In order to obtain a model-based estimate of the time constant of evidence integration, we fitted shifted exponential functions [40] to the d’ transformed psychometric functions of the LCA model fits of each observer. The shifted exponential function has been shown to accurately track the stimulus sensitivity increase as a function of time of human observers [40]. Fitting shifted exponential functions to the simulated (rather than the measured) d’ vs. duration functions provided more robust time constant estimates (Fig 4) by discounting noise in the behavioral data. We applied this procedure after ensuring that the LCA provided a reasonable fit to the behavioral (proportion correct) data of each subject (MLE, χ^2^ and *p* values for assessment of goodness of fit). We fitted the following shifted exponential function to the predicted average sensitivity across all coherences d' (*d*′ = Φ^-1^(0.99·*p*_*i*_)) vs. duration (Fig 4):

$$d^{'}\left(t\right)\;=\;D'(1-\text{exp}\left(-\frac{t-t0}{\tau }\right))$$ (10)

with *D′* denoting the average asymptotic sensitivity level for all coherences, *t*_*0*_ the period during which sensitivity was zero and *τ* the time constant (Fig 5).

## Results

We examined the impact of sensory-motor contingencies [41,42] on the timescale of perceptual evidence integration in a total of six human observers. To this end, we used a standard psychophysical task, coarse discrimination of visual motion direction, and quantified the integration timescale under variable (Fig 1A and 1B) or fixed (Fig 1C) DR-mapping. For the variable mapping, the mapping was instructed either before (“Pre”; Fig 1A) or after decision formation (“Post”; Fig 1B). Subjects 1–3 were asked to perform all three mapping conditions, to establish the main result in terms of three independent within-subject comparisons. Subjects 4 and 5 were measured to replicate the results of the “Pre” vs. “Post” comparison. Subject 6 was measured to replicate the result of the variable (only “Post”) vs. fixed comparison.

### Overall performance

In all observers and experimental conditions, performance depended lawfully on both, the strength and the duration of the visual motion signal (see Fig 2 for an example observer). The proportion of correct choices was generally about chance-level (~0.5) for the lowest level of coherently moving dots (0.05) and about perfect (~1) for the highest coherence level (0.5). This was true for all stimulus durations, except for the shortest (150 ms), at which even the highest coherence did not yield perfect performance in some subjects. We quantified the dependency on motion coherence by fitting cumulative Weibull functions to the proportion correct data. The psychometric functions generally shifted leftwards with increasing stimulus duration, as reflected by the decrease of the threshold parameter (inverse of sensitivity; Fig 2), meaning that observers could integrate the motion information over time and even discriminate patterns with a low percentage of coherently moving dots. The thresholds reached less then 0.1 motion coherence in all observers for the longer durations (Fig 3). This decrease of psychophysical thresholds with stimulus duration is an index of temporal integration of stimulus information (see below).

To assess whether observers made more “non-perceptual” errors (i.e., choosing the incorrect buttons irrespective of signal strength) in any of the three experimental conditions, we compared the “lapse rate” estimates (i.e., the upper asymptote of best-fitting cumulative Weibull functions) between conditions. Lapse rates quantify processes independent of the perceptual decision per se, such as lapses of attention or motor errors. Lapse rates were generally negligible in all three conditions (Table 1), and there were no systematic differences between the three conditions in most observers. One observer (subject 5) exhibited a significant difference in lapse rates between “Pre” and “Post” (p = 0.02), but also evident in the joint point (see below). There were also no differences in the overall threshold levels between the conditions when tested separately for all but the first (0.15 s) stimulus durations (Table 2).

**Table 1:** Lapse rates.

Subject	“Pre“	“Post“	“Fixed”
**1**	0.014 (0.000, 0.033)	0.000 (0.000, 0.004)	0.000 (0.000, 0.008)
**2**	0.000 (0.000, 0.000)	0.000 (0.000, 0.003)	0.000 (0.000, 0.000)
**3**	0.011 (0.000, 0.028)	0.000 (0.000, 0.000)	0.006 (0.000, 0.017)
**4**	0.049 (0.000, 0.070)	0.029 (0.030, 0.062)	-
**5**	0.052 (0.009, 0.070)	0.014 (0.000, 0.029)	-
**6**	–	0.000 (0.000, 0.000)	0.028 (0.000, 0.059)

Numbers are estimates of lapse rate (*λ*) and by 95% confidence intervals.

**Table 2:** Comparisons of threshold estimates between conditions.

Subject	Comparison	0.15s	0.30s	0.60s	1.2s	2.4s	4.8s
**1**	**Pre/Post**	0.356	0.002	0.079	0.174	0.204	0.284
	**Post/Fixed**	0.465	0.001	0.436	0.451	0.810	0.237
**2**	**Pre/Post**	**0.000**	0.277	0.002	0.163	0.374	0.363
	**Post/Fixed**	0.001	0.000	0.147	0.070	0.060	0.393
**3**	**Pre/Post**	0.004	0.447	0.311	0.090	0.316	0.401
	**Post/Fixed**	0.082	0.069	0.199	0.009	0.352	0.331
**4**	**Pre/Post**	0.005	0.293	0.175	0.332	0.403	0.414
**5**	**Pre/Post**	**0.000**	0.004	0.183	0.403	0.393	0.378
**6**	**Post/Fixed**	**0.000**	0.005	0.004	0.230	0.005	0.304

Numbers are p-values based on two-sided permutation tests. Significant p-values (after Bonferroni-correction for multiple comparisons) are printed in bold.

In sum, all six observers exhibited a high sensitivity and were able to adjust the sensory-motor associations and even rapidly (on a trial-by-trial basis) under the variable DR-mappings. There were no systematic differences in the occurrence of motor errors, as well as in overall performance, between the three conditions. Finally, the lawful shift in psychometric functions with stimulus duration indicates that observers indeed integrated stimulus information over time and thus became better in discriminating motion directions with low coherences the longer the observation period [12,13,26–28]. We next quantified the timescale of this integration process, and its dependence on DR-mapping, based on two complementary approaches: a model-independent and a model-based approach.

### Model-independent characterization of integration timescales

The model-independent approach was based on the following rationale. The integral of the “signal” embedded in the noisy stimulus increases linearly with the stimulus duration, while the “noise” (i.e., standard deviation) increases with the square root of the stimulus duration. Consequently, the signal-to-noise ratio increases with the square root of duration and perfect integration of the sensory evidence implies that the observers’ psychophysical threshold (the inverse of their signal-to-noise ratio) decreases as a function of the square root of stimulus duration, yielding a straight line with a slope of -0.5 on log-log axes. Conversely, no integration of sensory evidence implies that the observers’ threshold does not change as a function of stimulus duration (i.e., a straight line with a slope of 0). Consequently, integration of evidence across a limited time window predicts an initial linear decrease of the threshold vs. duration function with a slope of -0.5, followed by gradual deceleration towards a second (asymptotic) linear portion with a slope of 0. Note that such a timescale limitation may be due to leaky integration [37,38], or perfect (i.e., without leak) integration towards absorbing bounds [28] (see Discussion). Based on this rationale, the duration situated in between the -0.5- and 0-slope portions of the threshold vs. duration function can be used as an estimate of the integration timescale. We fitted bilinear functions to the individual threshold vs. duration functions, whereby the slope of the first line was constrained to -0.5 and the slope of the second to 0 and used the joint point between both lines as time scale estimate.

In all three conditions, the threshold vs. duration functions decreased with stimulus duration, but only for a limited range (Fig 3). The individual joint points ranged from 300 to 870 ms for “Pre” (presumably involving the highest short-term memory demands, see Discussion), from 300 to 610 ms for “Post”, and from 310 to 580 ms for “Fixed”. Any two frames of one of the three interleaved sequences of coherent motion were separated by 30 ms (at the monitor refresh rate of 100 Hz; see Materials and Methods), across which the observers’ visual motion system could pair dots to extract motion. Thus, the physical evidence fluctuated over 30 ms. Under the assumption that visual cortical regions like MT that encode visual motion track this stimulus information with high temporal precision [43], the shortest integration timescale observed here (300 ms) implies integration of ten samples of sensory evidence provided by visual cortex into the decision. The longest timescale of 870 ms corresponds to integration of close to 30 samples of sensory evidence.

Given the experimental design and fitting procedure, possible estimates of the joint point were confined to the interval 0.3–2.4 s (see Materials and Methods). All joint point estimates in the insets Fig 3 were shorter than the upper bound of 2.4 s, and the 95% confidence intervals of these joint point also estimates excluded 2.4 s. Thus, in all cases were the timescale significantly shorter than the one that would have maximized performance in this task entailing the maximum stimulus duration of 4.8 s.

In sum, all subjects showed temporal integration of perceptual evidence, but their integration timescale were consistently smaller than the optimal timescale for this task (defined as the timescale that would have maximized the overall fraction of correct choices). Possible mechanistic accounts of the timescale limitation are described in Discussion. We next explored if and how the timescale was affected by our manipulations of the sensory-motor contingencies.

#### Integration timescale for “Pre” vs. “Post” under variable DR-mapping

If the integration timescales depended on subjects’ ability to directly translate the integrated evidence into an action plan, then precluding the sensory-motor contingency in the “Post”-condition might be expected to shorten the integration timescale, relative to the “Pre”-condition. We found no consistent evidence for this scenario. Only one observer (subject 5 who also had a difference in lapse rates; Table 1) exhibited a significant difference in joint points between “Pre”- and “Post”-conditions (p < 0.01; two-tailed permutation test). In the remaining four observers (subjects 1–4) the joint points did not differ significantly between “Pre”- and “Post”-(range of p-values: 0.07–0.37; two-tailed permutation test).

Separate bi-linear fits with relaxed constraints on the two slopes (see Materials and Methods) yielded qualitatively identical results. The joint point estimates obtained from this procedure were generally less precise (larger confidence intervals, data not shown). Importantly, however, there was again no robust difference between joint points from the different conditions in all of the five observers (range of p-values across subjects 1–5: 0.09–0.77; two-tailed permutation test). Taken together, the results suggest that the evidence integration timescale is largely independent of the “Pre” vs. “Post” condition.

#### Integration timescale for fixed vs. variable DR-mapping

In previous studies reporting neural signatures of evidence integration in brain regions involved in motor planning [12,19–22,44,45], subjects were typically practiced with one specific sensory-motor contingency for at least hundreds of trials. It is possible that sensory-motor contingencies only improve the integration timescale after extensive practice, due to slow learning mechanisms. The trial-to-trial variation of DR-mapping in the “Pre-”condition of the previous experiment may have not have enabled such learning and, therefore, no improvement in integration timescale. To test this idea, we next explored the effect of long-term practice of one specific fixed DR-mapping on the integration timescale (Fig 1C), and compared this with the timescale under the variable DR-mapping.

The joint points obtained from the constrained fits (first slope: -0.5, second slope: 0) were statistically indistinguishable between the “Fixed” and “Pre” (range of p-values across three observers: 0.07–0.19; two-tailed permutation test) and the “Fixed” and “Post” conditions (range of p-values across four observers: 0.31–0.70; two-tailed permutation test) and. The bi-linear fits with relaxed constraints on the slopes yielded similar results: There was no significant difference in the joint points for two out of three observers in the “Fixed” vs. “Pre” comparison (subject 1: p = 0.81; subject 2: p = 0.02; subject 3: p = 0.21) and three out of four observers in the “Fixed” vs. “Post” comparison (subject 1: p = 0.41; subject 2: p = 0.68, subject 3: p = 0.66; subject 6 p < 0.01. Thus, even extensive practice of a specific sensory-motor association across several thousands of trials did not seem to robustly improve the temporal integration process.

#### Model-based characterization of integration timescales

One concern may be that our model-independent assessment of integration timescale may not have been sufficiently sensitive to reveal subtle effects of the sensory-motor contingencies. To address this, and to obtain a more theoretically motivated estimate of the integration timescale, we used a computational model of the decision process to fit the behavioral performance data (see Materials and Methods). The LCA is a neurophysiologically inspired model of the collective dynamics of two populations of “decision neurons”, which has been successfully applied to behavioral data from a wide variety of decision tasks [2,37,38,40]. For our interrogation protocol, the model assumed that the observers kept integrating for the whole stimulus interval and made a decision in favor of the alternative with the largest integrated evidence after that interval. Limitations of integration time scale resulted from the balance between “leaking away” of past evidence (biasing choices towards the most recent evidence) and mutual inhibition (biasing choices towards the early evidence).

The LCA model provided a reasonable fit to the behavioral performance of all observers in all three experimental conditions (Table 3). The goodness of fit was assessed by means of comparing the empirical and model-predicted psychometric functions using the chi-square statistic (see Materials and Methods). For an alpha value of 0.05, the null hypothesis that the two compared distributions (e.g. psychometric functions) are the same was not rejected for any of the participants (*p* >0.95 for all participants, see Table 3).

**Table 3:** LCA model parameters and goodness of fit.

Condition	Subject	*β*	*κ*	*s*	*I*_*0*_	*m*	*-LL*	*c* ^*2*^ *(*1, *N* = 29)	*p*
“Pre”	**1**	0.282	0.133	1.528	0.646	0.837	73.041	3.78	1.000
	**2**	0.497	0.020	1.696	-0.021	0.806	74.444	7.70	1.000
	**3**	0.436	0.028	2.379	-0.055	0.767	70.6333	7.17	1.000
	**4**	0.270	0.557	1.909	-0.064	0.381	88.095	6.03	1.000
	**5**	0.359	0.006	0.929	0.129	0.883	100.468	10.87	0.999
“Post”	**1**	0.126	0.090	2.403	0.507	0.985	61.137	4.33	1.000
	**2**	0.019	0.036	2.613	0.858	1.033	62.749	4.69	1.000
	**3**	0.061	0.063	9.181	1.125	1.371	40.263	2.06	1.000
	**4**	0.173	0.123	0.532	0.818	0.591	96.489	15.19	0.989
	**5**	0.257	0.110	2.178	0.484	1.083	76.182	6.17	1.000
	**6**	0.772	0.002	1.680	-0.237	0.732	61.299	3.79	1.000
“Fixed”	**1**	0.155	0.084	1.025	0.403	0.728	83.117	17.68	0.951
	**2**	0.179	0.011	1.622	-0.136	0.663	74.168	4.86	0.999
	**3**	0.176	0.093	1.449	0.456	0.672	65.839	8.25	0.999
	**6**	0.447	0.046	2.739	0.118	1.012	64.579	10.80	0.999

Parameters: inhibition *β*, leak *k*, sensitivity *s*, baseline activity (*I*_0_) and coherence saturation (*m*). See Materials for description of the meaning of these parameters.-*LL*, *χ* ^2^ **and *p*** are the negative log likelihood of the best-fitting parameters, the chi-square value and the p-value respectively.

We used the model with the best-fitting parameters for each observer to generate accuracy (d') vs. duration functions and fitted the average (across coherence levels) sensitivity with a shifted exponential function (Fig 4). The LCA fit of subject 4 in the “Pre”-condition was invariant to increases of duration and thus meaningful exponential fits could not be obtained. For the remaining subjects and conditions the obtained time constants were typically around 200 ms (Table 4, Fig 5B), consistently shorter than the around 500 ms timescale estimates obtained from the bi-linear fits (compare Fig 5A and 5B). This difference is expected since the time constant of the exponential fit represents the time it takes for the sensitivity to reach ~62% of its asymptotic value while the joint point indicates the exact moment of asymptotic saturation; by simulating a leaky integrator model we confirmed that the bilinear joint point is expected to be 2–2.5 times higher than the exponential time constant. The exact, analytical correspondence between the two measures of integration timescale should be addressed in future theoretical work. Despite the quantitative difference between the two timescale measures, there was a consistent qualitative correspondence across subjects (compare insets in Fig 3 with Table 4).

**Table 4:** Model-based time constant estimates.

Subject	“Pre”	“Post“	“Fixed”
**1**	0.151 (0.112, 0.212)	0.100 (0.038, 0.162)	0.093 (0.017, 0.169)
**2**	0.255 (0.221, 0.314)	0.197 (0.145, 0.251)	0.195 (0.152, 0.237)
**3**	0.170 (0.142, 0.192)	0.305 (0.251, 0.360)	0.178 (0.146, 0.210)
**4**	N/A	0.141 (0.087, 0.196)	—
**5**	0.190 (0.124, 0.255)	0.111 (0.032, 0.190)	—
**6**	—	0.273 (0.137, 0.409)	0.184 (0.124, 0.243)

Numbers are estimates of time constant (*τ*) and 95% confidence intervals.

Most importantly, in line with the analysis of joint points, comparison of the model-based integration time constants between conditions did not yield any significant difference between DR-mapping contexts, as assessed by comparing the 95% confidence intervals between conditions (Table 4).

**Fig 6:**
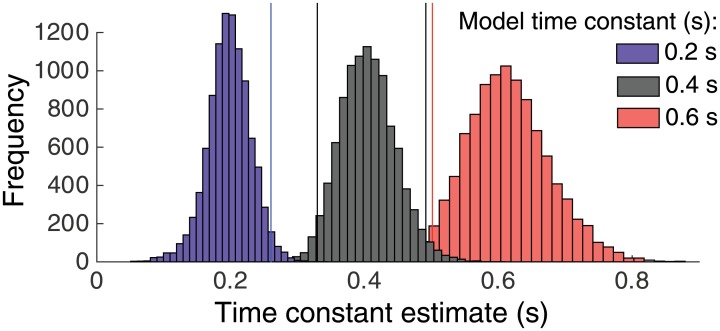
Sensitivity of the model-based timescale estimation. Sensitivity of the model-based timescale estimation. Three distributions of estimated time constants obtained by simulating leaky integrator models with three different time constants (0.2, 0.4, 0.6 s). Vertical lines indicate the 2.5% and 97.5% quantiles. The overlap between the distributions is small (<5%).

To assess the sensitivity of the model-based time constant estimation, we simulated a leaky integration model to generate many psychometric functions and repeated the same time constant estimation procedure (i.e., based on exponential fits as described in Materials and Methods) on these psychometric functions. In a simple leaky integration model, the integration time constant is known precisely (inverse of the leak parameter), whereas the time constant is a combination of leak and mutual inhibition in the more complex non-linear LCA described above (see Materials and Methods and [37]). We used three different levels of leak corresponding to time constants of 200, 400, and 600 ms, whereby 200 ms corresponds roughly to the median time constant estimate obtained from our behavioral data in all conditions (Fig 5B). Using three motion coherence levels (7%, 11%, 15%) and six stimulus durations (as in our behavioral experiments) we generated 300 simulated model responses per condition and repeated the time constant estimation process 10,000 times, for each of the three different time constants. The 95% confidence intervals of the resulting distributions of estimated time constants did not overlap (Fig 6). We conclude that our estimation procedure was sufficiently sensitive for distinguishing differences in time constants of 200 ms.

## Discussion

Here, we examined how knowledge of sensory-motor contingencies [41,42] affects the timescale of perceptual evidence integration into visual motion discrimination decisions, on a short-term (trial-by-trial) or long-term (across thousands of trials) basis. We found that all subjects integrated visual motion signals across time, and that their behavioral performance was well accounted by a leaky competing accumulator [37] model. However, integration timescales were generally shorter than optimal for the task (median joint points of ~450 ms), in which subject had to integrate evidence for up to 4.8 s. Finally, we found that neither subjects’ knowledge of a (variable) sensory-motor contingency, nor their long-term learning of one (fixed) contingency, had a significant and consistent effect on their integration timescale. Although we used two independent analytic approaches (model-independent and model-based) for estimating integration timescales which yielded consistent results, and established the sensitivity of our analyses, there was no evidence for a difference in integration timescales between the experimental conditions. We, therefore, conclude that long-term learning (under fixed mapping) or rapid acquisition (under variable mapping) of sensory-motor contingencies have only a small, if any, effect on the mechanisms limiting the timescale of perceptual decisions.

Many studies have used free response protocols, in which the observer controls the decision time, to study the dynamics of perceptual decision-making [8,9,31,37,46]. Evidence integration is then inferred from fitting a decision model to the reaction time distributions [2,3,8,37]. Reaction times do not only reflect the integration process, but also the observer’s speed-accuracy tradeoff [3,6,8,9,47]. By contrast, in the interrogation protocol used in the present study, the optimal strategy is to integrate all available evidence (perhaps subject to inevitable leak), and then choose the option that is best supported by the integrated evidence when the response is prompted [3,37]. Thus, the improvement of threshold as a function of stimulus duration should directly reflect the evidence integration process.

Previous studies of perceptual decision-making in humans and animals using this approach yielded a wide range of integration timescales. Some studies of human motion discrimination found timescales on the order of several seconds [26,27]. One study in rats and humans showed close-to-perfect integration, within a range of ~1 s using a task where participants had to discriminate the relative frequency of discrete events [29]. Another human study showed threshold decreases throughout a range of 900 ms—crucially, this decrease was steeper when subjects expected longer signals compared to when they expected shorter signals [13]. The relatively short timescales observed in the current study are in line with previous results from monkeys [28] in motion discrimination: In particular, using stimuli and tasks analogous to our fixed DR-mapping condition, Kiani et al. [28] found that monkeys exhibited a joint point of ~420 ms, just like our current human results. The human studies with larger samples sizes performing the same task also observed a substantial inter-individual variability in integration timescales [13]. Taken together, these results may suggest that integration timescale may, just like short-term memory capacity, be an individual trait with an upper limit, which can only be adapted to task demands within that limited range [13]. Additionally, the length of the temporal integration window may differ across different tasks.

Limited integration timescales are consistent with two mechanistically distinct scenarios. First, the decision process may terminate prematurely, once the integrated evidence has reached an implicit absorbing bound (termed “bounded diffusion”) [28]. This strategy can be compared to “closing the eyes” after an initial decision is made, thus eliminating the impact of the subsequently presented evidence. Second, the limit may be explained using a leaky competing accumulation process, as postulated by the LCA model that we used here [38,48]. If the leak parameter exceeds inhibition then the model implements a stable Ornstein-Uhlenbeck (leaky integration) process, with integration taking place only until the stable state is reached [3]. When the inhibition parameter is larger than leak, the model has unstable dynamics and is maximally sensitive to early evidence, in close resemblance to the bounded diffusion model. Both the implicit boundary and LCA dynamics might also work in concert, and to different extent in different individuals. The current findings establish that, whatever the mechanism limiting the integration time scale, this mechanism seems unaffected by sensory-motor mapping.

The variable DR-mapping forced observers to establish and remember a new association between decision and response on each trial, whereas the fixed mapping allowed them to establish an automatic sensory-motor transformation, presumably after a few hundred trials of practice [49]. Specifically, maintaining a new DR-mapping rule online during the decision formation (“Pre”-condition) increased short-term memory load [49,50], which may have interfered with the integration process. This difference in task demand seemed not to affect the behavioral performance measures–neither the mean integration timescales, nor the frequency of simple motor errors (lapse rates; Table 1).

Our results have a number of implications for neurophysiological studies of perceptual decision-making. First, several studies into the neural basis of decision-making have used a manipulation analogous to our “Post-condition” to decouple decision-making from action planning [12,23,42,51–54]. Our present results indicate that these studies, in fact, probe decision dynamics analogous to those occurring in the classical tasks with fixed mapping. Second, our observation of limited integration time scales question an assumption that has been implicit in several fMRI and neurophysiological studies using interrogation protocols [16,20,21,30,50,53–55]: that observers integrate all sensory information provided, even for extended (> 1 s) stimulus durations. Our results indicate that this assumption should be verified for each experimental condition and subject. Finally, our findings shed new light on the build-up activity commonly observed during decision formation in motor structures of the human brain [21,22,23,45,56]: Albeit providing a useful neural marker of the evolving integration process, this activity seems to be a downstream consequence of the integration process rather than a direct correlate of that process. Our results are consistent with a growing body of physiological evidence [23,53,57–59] indicating that evidence integration during decision-making is distinct from action planning.
