# Correction: Action Planning and the Timescale of Evidence Accumulation

**DOI:** 10.1371/journal.pone.0133332

**Published:** 2015-07-15

**Authors:** Konstantinos Tsetsos, Thomas Pfeffer, Pia Jentgens, Tobias H. Donner

There are errors in the Funding section. The correct funding information is as follows:

This work was funded by the Amsterdam Brain and Cognition priority program; grant number: ABC2014-01 (http://abc.uva.nl/research/nav), the European Union 7th Framework Programme (FP7/2007-2013) under grant agreement no. 604102 (Human Brain Project; http://www.humanbrainproject.eu), and the Deutsche Forschungsgemeinschaft (DFG), SFB 936/Z1 (http://www.sfb936.net). These funders had no role in study design, data collection and analysis, decision to publish, or preparation of the manuscript.
